# Rapid specialization of counter defenses enables two-spotted spider mite to adapt to novel plant hosts

**DOI:** 10.1093/plphys/kiab412

**Published:** 2021-08-31

**Authors:** Golnaz Salehipourshirazi, Kristie Bruinsma, Huzefa Ratlamwala, Sameer Dixit, Vicent Arbona, Emilie Widemann, Maja Milojevic, Pengyu Jin, Nicolas Bensoussan, Aurelio Gómez-Cadenas, Vladimir Zhurov, Miodrag Grbic, Vojislava Grbic

**Affiliations:** 1 Department of Biology, The University of Western Ontario, London, Ontario N6A 5B8, Canada; 2 Instituto de Ciencias de la Vid y el Vino (CSIC, UR, Gobiernode La Rioja), Logrono 26006, Spain; 3 Departament de Ciències Agràries i del Medi Natural, Universitat Jaume I, Castelló de la Plana, E-12071, Spain; 4 Department of Biology, University of Belgrade, Belgrade, Serbia

## Abstract

Genetic adaptation, occurring over a long evolutionary time, enables host-specialized herbivores to develop novel resistance traits and to efficiently counteract the defenses of a narrow range of host plants. In contrast, physiological acclimation, leading to the suppression and/or detoxification of host defenses, is hypothesized to enable broad generalists to shift between plant hosts. However, the host adaptation mechanisms used by generalists composed of host-adapted populations are not known. Two-spotted spider mite (TSSM; *Tetranychus urticae*) is an extreme generalist herbivore whose individual populations perform well only on a subset of potential hosts. We combined experimental evolution, *Arabidopsis thaliana* genetics, mite reverse genetics, and pharmacological approaches to examine mite host adaptation upon the shift of a bean (*Phaseolus vulgaris*)-adapted population to Arabidopsis. We showed that cytochrome P450 monooxygenases are required for mite adaptation to Arabidopsis. We identified activities of two tiers of P450s: general xenobiotic-responsive P450s that have a limited contribution to mite adaptation to Arabidopsis and adaptation-associated P450s that efficiently counteract Arabidopsis defenses. In approximately 25 generations of mite selection on Arabidopsis plants, mites evolved highly efficient detoxification-based adaptation, characteristic of specialist herbivores. This demonstrates that specialization to plant resistance traits can occur within the ecological timescale, enabling the TSSM to shift to novel plant hosts.

## Teaser

Mites can evolve highly efficient detoxification-based adaptation in approximately 25 generations on an initially unfavorable plant host, revealing that specialization can occur within the ecological timescale.

## Introduction

During millions of years of co-evolution with their host plants, herbivores have developed two main strategies to counteract plant resistance traits. Specialist herbivores have evolved highly efficient adaptation mechanisms against a limited set of host defenses, including modified feeding behavior (Helmus and Dussourd, 2005), suppression of plant defenses (Zhao et al., 2015), reduced xenobiotic target site sensitivity (Dobler et al., 2012), sequestration (Beran et al., 2018), and detoxification of plant toxins (Ratzka et al., 2002). In contrast, generalist herbivores evolved an innate ability to feed on a broad range of host plants that display a wide array of resistance traits (Despres et al., 2007; Barrett and Heil, 2012; Heidel-Fischer and Vogel, 2015). The generalist lifestyle is considered to be ecologically and evolutionarily advantageous (Futuyma and Moreno, 1988; Loxdale and Harvey, 2016). However, the fitness tradeoffs arising from the biochemical and physiological costs of polyphagy have been proposed to select for the specialization of herbivore–plant interactions (Dall and Cuthill, 1997). Generalist herbivores evolved two main strategies to counteract a diverse array of plant host defenses. Broad generalists, where all individuals can feed on a wide range of plant hosts, rely on rapid transcriptional plasticity to colonize distantly related plants (Fenton et al., 1998; Mathers et al., 2017). However, the majority of generalist herbivores are regarded as composites of populations that themselves thrive on a subset of potential hosts (Fox and Morrow, 1981; Peccoud et al., 2009; Barrett and Heil, 2012). These populations exist in a wide range of host specializations and are proposed to ultimately lead to the formation of specialist species (Peccoud et al., 2009). Consistent with these theoretical predictions, specialist herbivores dramatically outnumber generalists (Rafter and Walter, 2020). Specialization to plant resistance traits is assumed to occur over a long evolutionary time and evokes complex adaptive genetic changes that result in the establishment of highly efficient adaptation mechanisms against a limited set of host defenses. Despite our understanding of host specialization in specialist herbivores, the mechanisms that mediate the formation of host-adapted populations in generalist herbivores are poorly understood.

Attenuation of plant responses induced by herbivore feeding has been proposed to be one of the mechanisms of host adaptation (Zarate et al., 2007; Kant et al., 2008; Musser et al., 2012; Wu et al., 2012). Consistently, effectors that modulate plant defenses have been identified in secretions of a number of generalist herbivores belonging to different feeding guilds (Musser et al., 2002; Hogenhout and Bos, 2011; Wu et al., 2012; Bass et al., 2013; Jonckheere et al., 2016; Kaloshian and Walling, 2016; Basu et al., 2018). Their mode of action is largely unknown but, to be effective against many host plants, it is assumed that they either target conserved compounds or pathways associated with plant defense or have a broad-spectrum activity with only a specific subset being effective against any particular host. Another general mechanism of host adaptation is metabolic resistance whereby herbivores effectively detoxify ingested plant toxins. In specialists, metabolic resistance is based on a limited number of detoxification enzymes that have high specificity and efficiency for a given plant toxin (Ratzka et al., 2002; Li et al., 2003; Wittstock et al., 2004; Mao et al., 2006; Gloss et al., 2014; Heidel-Fischer et al., 2019). Genes encoding these enzymes usually carry nucleotide substitutions in coding regions that increase their activity against the plant toxin (Wen et al., 2006; Dobler et al., 2012; Gloss et al., 2014) and/or in the promoter sequences resulting in their constitutive and high level of expression (Bass et al., 2013). In contrast, it is assumed that generalist herbivores rely on ubiquitous classes of detoxification enzymes (e.g. carboxyl/cholinesterases, cytochrome P450 monooxygenases (P450s), glutathione S-transferases (GSTs), UDP-glycosyltransferases (UGTs), and ABC transporters) that were shown to accept structurally diverse substrates which they metabolize with low levels of activity (Li et al., 2004; Halon et al., 2015; Shi et al., 2018; Snoeck et al., 2019). Genes encoding general detoxification enzymes have undergone extensive amplification and neofunctionalization, and are transcriptionally responsive to a wide range of xenobiotics (Govind et al., 2010; Grbic et al., 2011; Zhurov et al., 2014; Wybouw et al., 2015; Muller et al., 2017; Schweizer et al., 2017). Thus, it is hypothesized that attenuation of plant defenses combined with the expanded and functionally versatile detoxification capabilities enable generalist herbivores to cope with diverse allelochemicals and to feed on many host plants.

The two-spotted spider mite (TSSM, *Tetranychus urticae*) is an extreme generalist herbivore that feeds on over 1,100 plant species from more than 100 families (Migeon and Dorkeld, 2021). Such a wide host range indicates that TSSM can counteract a great diversity of plant resistance traits. However, individual TSSM populations do not perform equally well on all potential host plants (Fry, 1989; Agrawal et al., 2002; Fellous et al., 2014). Instead, TSSM has an outstanding ability to adapt to new hosts (Gould, 1979; Fry, 1989; Magalhaes et al., 2007; Wybouw et al., 2015). The mechanism of this host adaptability is not known. The analysis of plant transcriptional changes following the host shift revealed that some TSSM populations can suppress plant-induced responses (Kant et al., 2008; Wybouw et al., 2015). At the same time, TSSM massively reprograms its detoxification capacity (Dermauw et al., 2013; Zhurov et al., 2014; Wybouw et al., 2015) and the complement of its salivary secretions (Villarroel et al., 2016; Jonckheere et al., 2016, 2018). However, no functional evidence explains whether these changes contribute to TSSM host adaptation or if they merely reflect stress responses or different feeding physiology due to the host shift. In addition, it remains unclear if changes to the initial xenobiotic responses are sufficient for TSSM adaptation to a new host, or if additional changes are required for the evolution of TSSM host adaptation.

Even though plants belonging to the Brassicaceae family have been recorded as TSSM plant hosts (Migeon and Dorkeld, 2021), *Arabidopsis thaliana* is a nonpreferred host for the TSSM London reference population that is reared on bean (*Phaseolus vulgaris*; Zhurov et al., 2014). In an experimental evolutionary setup, we adapted the TSSM London ancestral population to Arabidopsis*.* Using functional pharmacological and reverse genetics experiments that independently suppressed the activities of families of mite detoxification enzymes, we provide in vivo functional evidence that TSSM requires cytochrome P450 activity for adaptation to Arabidopsis. We identified activities of two tiers of P450s: general xenobiotic-responsive P450s that have a limited contribution to mite adaptation to Arabidopsis and adaptation-associated P450s that efficiently counteract Arabidopsis defenses. Our data reveal that in approximately 25 generations, mites established a highly efficient detoxification response to Arabidopsis defenses, demonstrating that specialization to plant resistance traits can occur within an ecological timescale.

## Results

### TSSM feeding induces complex jasmonic acid-regulated Arabidopsis defenses

**Figure 1:**
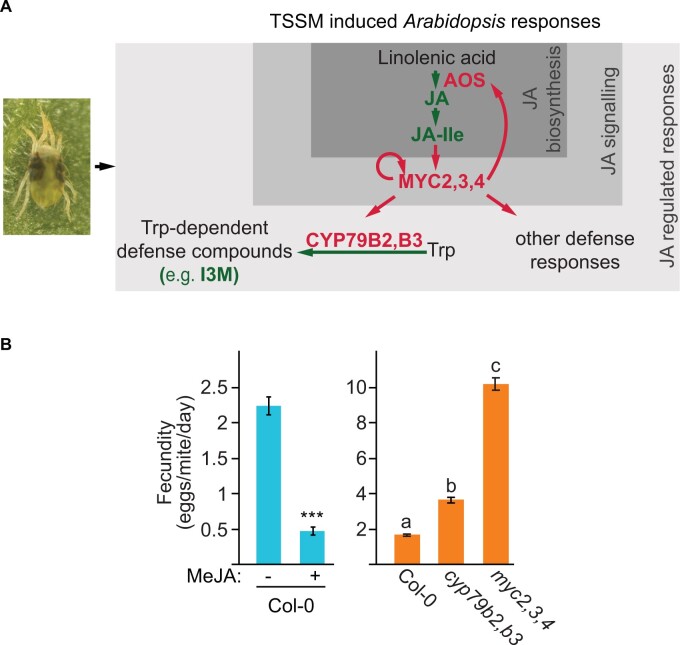
TSSM (*T. urticae*) induces complex defense responses in Arabidopsis. A, A simplified schematic of TSSM feeding-induced Arabidopsis defense responses. AOS, ALLENE OXIDE SYNTHASE; JA, jasmonic acid; JA-Ile, jasmonoyl-isoleucine; CYP79B2,B3, CYTOCHROME P450 FAMILY 79 SUBFAMILY B POLYPEPTIDE 2 and 3, respectively; Trp, tryptophan; MeJA, methyl jasmonate. B, The fecundity of bean-reared TSSM (ancestral population) upon feeding on MeJA treated Col-0 plants (left) and on Col-0 wild type, *cyp79b2 cyp79b3* and *myc2 myc3 myc4* mutant plants (*myc2,3,4*) (right). Data are presented as mean number of eggs laid by a female mite per day ± sem (standard error of the mean). Left, *n* = 18; asterisks indicate a significant difference between treated and control samples (ANOVA: ***P < 0.001). Right, *n* = 15 Different letters represent significant differences between means (Tukey’s HSD test, *α* = 0.05).

To understand the mechanism of TSSM adaptation to Arabidopsis, we first characterized the complexity of Arabidopsis defenses that mites have to overcome in order to use Arabidopsis as a host. Jasmonic acid (JA) and its bioactive conjugate jasmonoyl-isoleucine (JA-Ile), Figure 1A, were shown to be required for Arabidopsis defenses against TSSM herbivory (Zhurov et al., 2014). A dramatic five-fold decrease of mite fecundity on methyl jasmonate (MeJA)-treated Columbia-0 (Col-0) plants (Figure 1B), indicates that JA-induced defenses are sufficient for Arabidopsis resistance against TSSM. JA responsiveness is mediated by the MYC2, MYC3, and MYC4 (MYC2–4) transcriptional activators (Devoto et al., 2005; Fernandez-Calvo et al., 2011), and consistently, *myc2,3,4* mutant plants are extremely susceptible to TSSM (Figure 1B). Among the MYC2,3,4-regulated genes are *CYTOCHROME P450 FAMILY 79 SUBFAMILY B POLYPEPTIDE 2 and 3* (*CYP79B2 and CYP79B3*, respectively) that are required for the synthesis of tryptophan (Trp)-derived secondary metabolites (Figure 1A; Hull et al., 2000; Mikkelsen et al., 2000; Schweizer et al., 2013). One class of these metabolites are indole glucosinolates, shown to protect Arabidopsis plants against herbivory of a wide range of arthropods, including mites (Kim and Jander, 2007; Kim et al., 2008; Hopkins et al., 2009; Elbaz et al., 2012; Whiteman et al., 2011, 2012; Zhurov et al., 2014; Widemann et al., 2021). The modest increase in fecundity when TSSM fed on *cyp79b2 cyp79b3* (*cyp79b2,b3)* plants (Figure 1B) indicates that besides indole glucosinolates, there are other MYC2,3,4-regulated defenses that prominently protect Arabidopsis plants against mites. Thus, there are at least two distinct classes of JA-regulated Arabidopsis resistance traits against TSSM are *CYP79B2 CYP79B3-*dependent and *CYP79B2 CYP79B3-*independent.

### Mites can adapt to a complex array of Arabidopsis defenses

**Figure 2:**
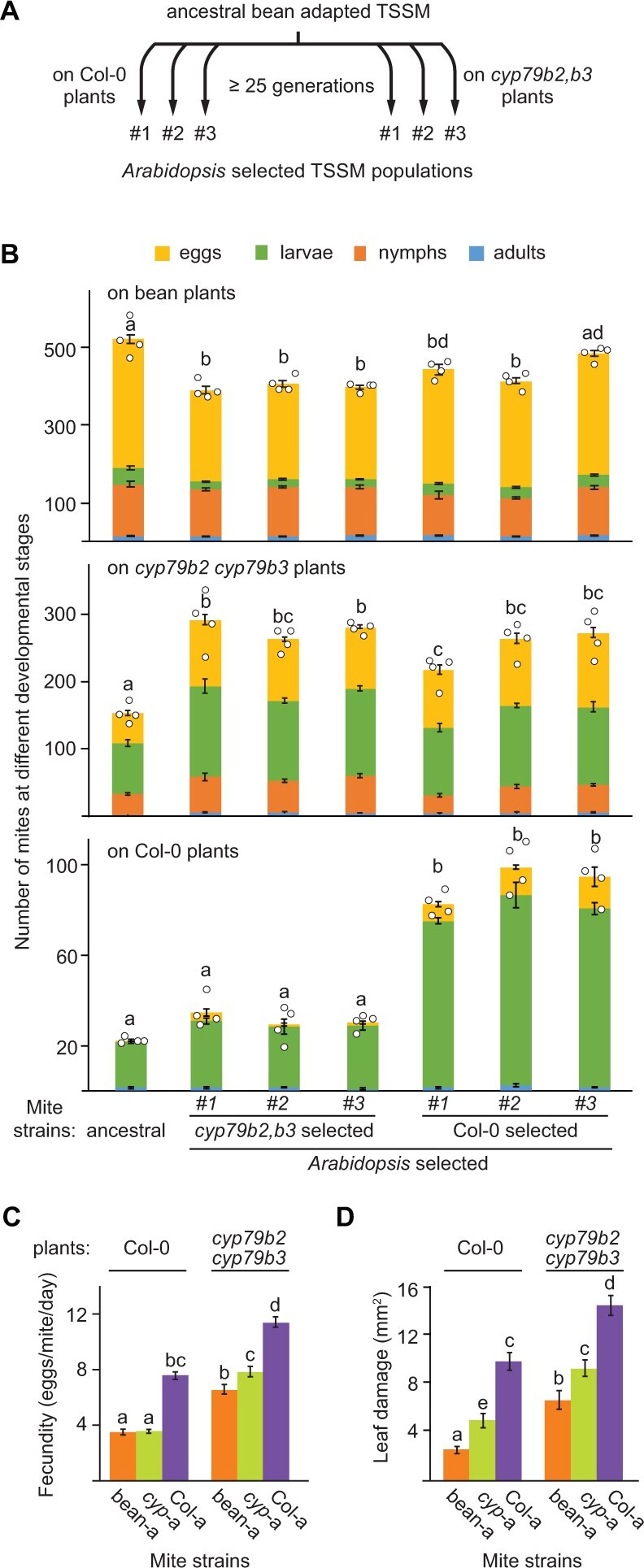
TSSM can adapt to a complex array of Arabidopsis-induced defenses. A, A schematic of the experimental evolution procedure. B, Performance of ancestral and selected populations on bean (top), *cyp79b2 cyp79b3* (middle), and Col-0 (bottom) plants. Data are from the indirect transfer experiment where Arabidopsis selected TSSM were reared for two generations on bean before transfer to the experimental plants. Data represent the mean size of the population (the sum of eggs, larvae, nymphs, and adults) derived from 20 adult female mites at 7-d postinfestation ± sem, *n* = 4. Different letters represent significant differences between means (Tukey’s HSD test, *α* = 0.05). Data from direct transfer experiments where Arabidopsis selected TSSM were directly transferred from their rearing to the experimental plants is shown in Supplemental Figure S1. C, Fecundity of the ancestral (bean-a) and populations #3 of Arabidopsis-selected mites (*cyp*-a and Col-a) upon feeding on Col-0 and *cyp79b2 cyp79b3* plants. Data are presented as mean number of eggs laid by a female mite per day ± SEM, *n* = 30. D, Leaf damage of Col-0 and *cyp79b2 cyp79b3* Arabidopsis plants upon herbivory of ancestral (bean-a), and populations #3 of Arabidopsis-selected mites (*cyp*-a and Col-a). Data represent mean area of chlorotic spots (mm^2^) ± sem, *n* = 18. C and D, Different letters represent significant differences between means (Tukey’s HSD test, *α* = 0.05). Individual sample values are shown as open circles for *n* ≤ 10.

To test if TSSM can adapt to the complex array of Arabidopsis defenses, we used an experimental evolutionary setup where an ancestral TSSM population that is highly susceptible to Arabidopsis defenses (Zhurov et al., 2014) was transferred and continuously maintained on Arabidopsis plants. The improvement of mite fitness on a new host has been reported to occur in as soon as 5–10 generations (Fry, 1989, 1990; Agrawal, 2000); however, the improvement is gradual and reaches a stable state after 15–20 generations, depending on the ancestral population and the plant host (Fry, 1989; Agrawal, 2000; Magalhaes et al., 2009). We initiated the analysis of mite adaptation after 18 months (≥25 generations) of selection (Figure 2A) to ensure consistency of the mite adaptation state throughout the analysis. We initially infested Arabidopsis plants with approximately 1,000 fertilized female mites in a triplicated experiment on two Arabidopsis genotypes: (1) Col-0, fully defended, wild-type plants, and (2) *cyp79b2 cyp79b3* mutant plants that lack Trp-derived compounds but display the remaining JA-regulated defenses (Figures 1, A and 2, A). The performance of the ancestral and selected mite populations was subsequently quantified by counting the total number of eggs, larvae, nymphs, and adults derived from 20 adult females in 7 d. The performance was measured in two experimental regimes: (1) direct transfer, where the initial mites were moved from their corresponding rearing plant hosts directly to the experimental plants and (2) indirect transfer, which included mite maintenance on bean plants for two generations before their transfer to the experimental plants. The performance of the ancestral and selected mite populations on bean, or Arabidopsis *cyp79b2 cyp79b3* and Col-0 plants had similar patterns in both direct (Supplemental Figure S1) and indirect (Figure 2B) transfer regimes, indicative of genetic adaptation that is independent of maternal and environmental physiological effects.

On bean plants (Figure 2B, top), Arabidopsis-selected mite populations had slightly but significantly lower performance relative to the ancestral population, indicating that TSSMs can evolve the ability to exploit different hosts without a major reduction in their performance on the ancestral plants. On *cyp79b2 cyp79b3* plants (Figure 2B, middle), the two Arabidopsis-selected mite populations performed significantly better than the ancestral population, suggesting that they can overcome *CYP79B2 CYP79B3*-independent Arabidopsis defenses to a similar extent. On Col-0 plants (Figure 2B, bottom), only mites selected on Col-0 had increased performance over the ancestral population, showing that Col-0 selected mites were also able to adapt to *CYP79B2 CYP79B3-*dependent defenses. However, mites selected on *cyp79b2 cyp79b3* plants and the ancestral mite population that were not exposed to *CYP79B2 CYP79B3*-dependent Arabidopsis defenses were susceptible to these defenses and had similar and low performances when they fed on Col-0 plants.

We arbitrarily chose populations #3 of *cyp79b2 cyp79b3* and Col-0 selected mites for subsequent studies that were all performed in indirect transfer regimes. The analysis of additional mite fitness parameters, fecundity, and Arabidopsis leaf damage caused by mite feeding (Figure 2, C and D, respectively) confirmed the adaptation status of these mite populations (from now on referred to as *cyp*-a (for #3 *cyp79b2 cyp79b3* selected mite population), Col-a (for #3 Col-0 selected mite population), and bean-a (for the ancestral bean-adapted [bean-a] London TSSM population). Thus, our data demonstrate that TSSM can adapt to novel plant hosts that have a complex array of defenses. Importantly, Arabidopsis-adapted mite populations retained high fitness on bean plants, thus, they expanded their host range without losing the ability to feed on the ancestral host. Moreover, mite adaptation to *CYP79B2 CYP79B3*-independent Arabidopsis defenses (present in both types of Arabidopsis-selected mite populations) can be uncoupled from mite adaptation to Trp-derived defenses (present only in mites selected on Col-0 plants).

### Ancestral and Arabidopsis-adapted mite strains induce similar JA-regulated Arabidopsis defenses

**Figure 3:**
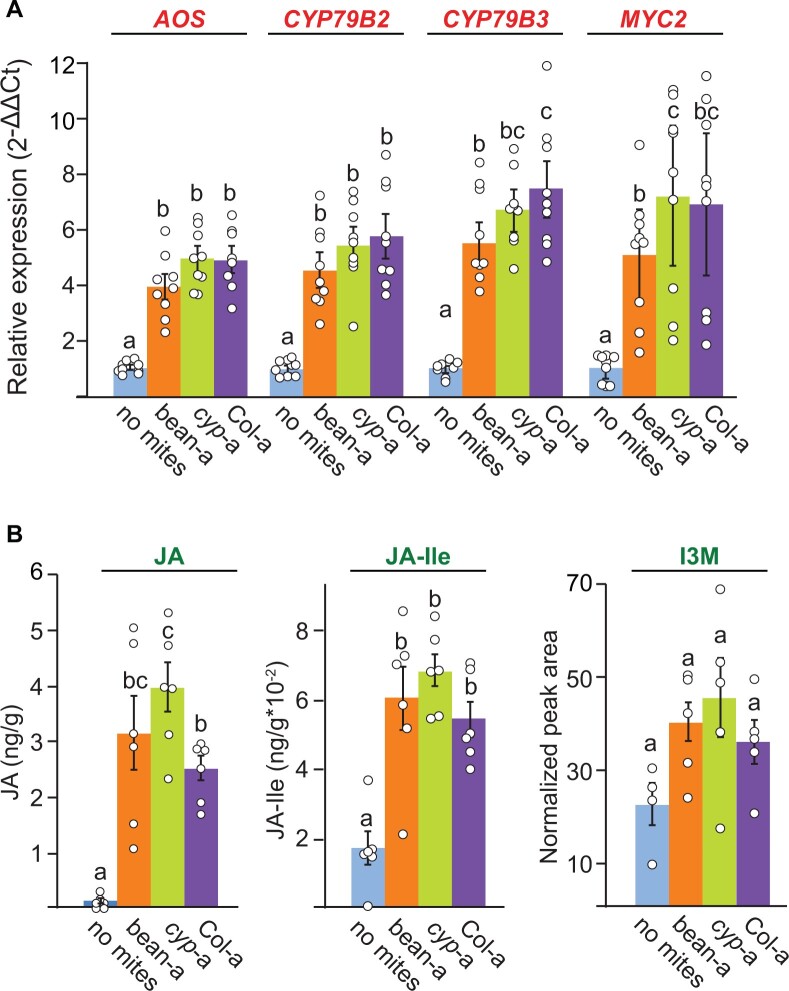
Bean-a, *cyp*-a, and Col-a mites induce similar Arabidopsis defense responses. A, Expression of *AOS, CYP79B2, CYP79B3*, and *MYC2* genes in Col-0 leaves in response to bean-a, *cyp*-a, and Col-a mite feeding. Shown are means ± SEM of fold changes of expression levels detected by RT-qPCR relative to no mite control, *n* = 9. Primer sequences and amplification efficiencies (E) used in qPCR are shown in Supplemental Table S1. *PEROXIN4* (*AT5G25760*), was used as the reference gene. B, Levels of JA and JA-Ile (ng g^−1^ fresh weight), and relative level of the I3M (shown as normalized peak area), in 3-week-old Col-0 plants after herbivory of bean-a, *cyp*-a, and Col-a mites for 24 h. Values are means ± sem, *n* = 6. Different letters represent significant differences between means (Tukey’s HSD test, *α* = 0.05). Individual sample values are shown as open circles for *n* ≤ 10.

Suppression of induced plant defenses was proposed to be one of the mechanisms of TSSM adaptation to new host plants (Villarroel et al., 2016; Blaazer et al., 2018; Jonckheere et al., 2016, 2018). One of the hallmarks of defense-suppressing TSSM populations is the similarity of their performance on wild-type and defense-deficient host plants (Kant et al., 2008). However, Arabidopsis-adapted mites, like the ancestral population, performed significantly better on *cyp79b2 cyp79b3* than on Col-0 plants (Figure 2, B and C), suggesting that mite adaptation to Arabidopsis is not based on the suppression of host defenses. To corroborate the lack of defense suppression in Arabidopsis-adapted mites we determined the expression levels of JA-responsive *ALLENE OXIDE SYNTHASE* (*AOS), MYC2, CYP79B2, and CYP79B3* genes (labeled in red in Figure 1A) and the abundance of JA, JA-Ile, and indol-3-ylmethylglucosinolate (I3M) (labeled in green in Figure 1A) that were previously established as reliable markers of induced JA-regulated Arabidopsis defenses against mite herbivory (Zhurov et al., 2014; Widemann et al., 2021). The expression levels and the abundance of defense-associated markers were determined in Col-0 plants that were challenged with bean-a, *cyp*-a, or Col-a mites after 24 h of mite herbivory. As seen in Figure 3A, *cyp*-a and Col-a mites induced all JA-regulated marker genes to similar or higher levels relative to nonadapted bean-a mites. Likewise, JA, JA-Ile, and I3M accumulated at comparable levels in bean-a, *cyp*-a, and Col-a challenged Col-0 plants (Figure 3B). Even though we cannot rule out the possibility that some plant pathways with minor effects on mite fitness may have been suppressed, our data suggest that ancestral and Arabidopsis-adapted mites are exposed to similar JA-regulated Arabidopsis defenses, shown to be both necessary and sufficient to protect Arabidopsis plants against mite herbivory.

### Initial responses to Arabidopsis xenobiotics are similar in ancestral and Arabidopsis-adapted mites

**Figure 4:**
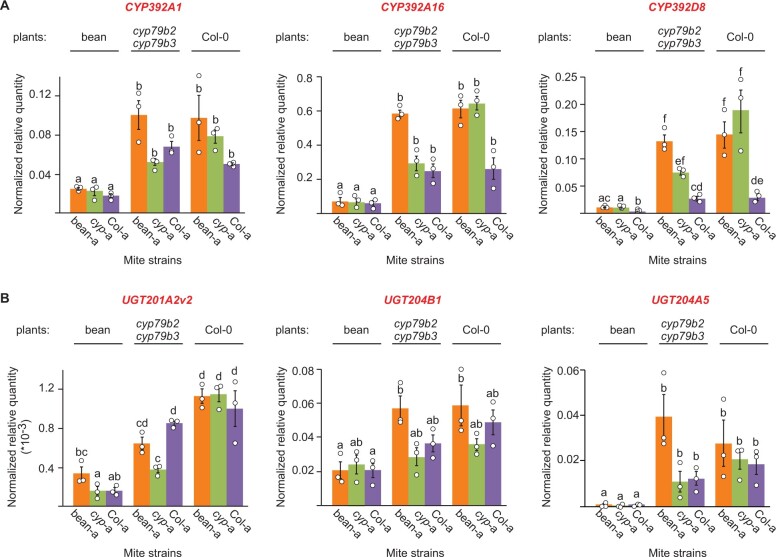
Levels of Arabidopsis-induced mite *cytochrome P450 and UGT* genes. A, *CYP392A1* (*tetur07g06410*), *CYP392A16* (*tetur06g04520*), and *CYP392D8* (*tetur03g05070*). B, *UGT201A2v2* (*tetur02g02480*), *UGT204B1* (*tetur02g09830*), and *UGT204A5* (*tetur05g00090*). Shown are means ± sem for relative quantity of expression normalized to the *RP49* (*tetur18g03590*) reference gene, *n* = 3. Primer sequences and amplification efficiencies (E) used in qPCR are shown in Supplemental Table S1. Different letters represent significant differences between means (Tukey’s HSD test, *α* = 0.05). Individual sample values are shown as open circles for *n* ≤ 10.

The constitutive overexpression and/or increased transcriptional plasticity of genes encoding xenobiotic-metabolizing enzymes have been previously implicated in the evolution of TSSM resistance to pesticides (Van Leeuwen et al., 2010; Kwon et al., 2015; Van Leeuwen and Dermauw, 2016) and mite adaptation to several plant hosts (Agrawal et al., 2002; Wybouw et al., 2012, 2014, 2015). We previously identified 40 genes, primarily encoding cytochrome P450s and UGT, that were associated with the initial TSSM responses to varying levels of Trp-derived defense compounds (Zhurov et al., 2014). To test if these genes may have contributed to mite adaptation to Arabidopsis, we arbitrarily chose three genes within each class *(CYP392A1, CYP392A16, CYP392D8, UGT201A2v2, UGT204B1*, and *UGT204A5)* and used reverse-transcription quantitative PCR (RT-qPCR) to determine their expression in bean-a, *cyp*-a, and Col-a mites that were kept on bean plants for two generations before they were transferred to bean and Arabidopsis plants with (Col-0) or without (*cyp79b2 cyp79b3*) Trp-derived defense compounds. On the bean plants, all marker genes had similar and low expression levels in both bean-a and Arabidopsis-adapted mites (Figure 4), indicating that none of the tested genes underwent constitutive upregulation during mite adaptation to Arabidopsis. Consistent with the previous report (Zhurov et al., 2014), the transfer of bean-a mites to *cyp79b2 cyp79b3* or Col-0 plants resulted in induced expression of all tested genes. Their expression was also induced in *cyp*-a and Col-a mites when they were shifted from bean to Arabidopsis plants. Even though there was variability in the relative expression of chosen *CYP and UGT* genes in these mites, their levels were either comparable or lower than in bean-a mites (Figure 4). Thus, neither higher constitutive expression nor greater transcriptional plasticity of tested *CYP and UGT* genes associates with the Arabidopsis-adapted mites, demonstrating that: (1) Arabidopsis-adapted mites retained responsiveness to Arabidopsis xenobiotics and (2) the expression of tested mite genes does not associate with TSSM adaptation to Arabidopsis*;* instead, it associates with general xenobiotic responsiveness of bean-a, *cyp*-a, and Col-a mites to shift from bean to Arabidopsis plants.

### Metabolic resistance underlies TSSM adaptation to Arabidopsis

To identify changes in the detoxification potential associated with mite adaptation to Arabidopsis, we determined global enzymatic activity for three main protein families—esterases, GSTs, and P450s—in ancestral and Arabidopsis-adapted mite populations feeding on bean and Arabidopsis plants. Overall, activities of all three enzymatic classes were responsive to the host plant challenge: they were lowest when mites fed on bean plants, and they progressively increased with the complexity of Arabidopsis defenses (Supplemental Figure S2). However, of the three classes of detoxification enzymes, P450 activity was consistently higher in Col-a mites across all plant hosts, indicating that both constitutive and inducible levels of P450 activity increased in Col-a mites (three-way analysis of variance (ANOVA); plant host: mite strain interaction *F* = 103.93, *P* = 5.451e-16, followed by Tukey’s honestly significant difference (HSD) test, *P* < 0.01, *n* = 4).

**Figure 5:**
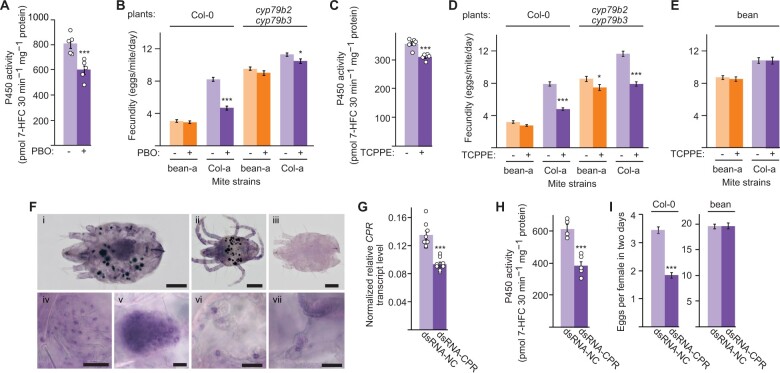
Cytochrome P450 activity is required for TSSM adaptation to Arabidopsis. A and C, The activities of cytochrome P450 in Col-a mites feeding on Col-0 plants after the application of PBO (A) and TCPPE (C) inhibitors of P450 activity. Data are represented as mean ± sem, *n* = 5. B and D, The effects of PBO (B) and TCPPE (D) on fecundity of bean-a and Col-a mites upon feeding on Col-0 and *cyp79b2 cyp79b3* plants. E, The effect of TCPPE treatment on fecundity of bean-a and Col-a mites upon feeding on bean plants. Fecundity in (B), (D), and (E) was measured over 6 d, and data are presented as mean number of eggs laid by a female mite per day ± sem; *n* = 30. F, Whole-mount in situ localization of *Tu-CPR* in TSSM (i, ii, iv–vii, anti-sense probe). (i) adult female, (ii) adult male, (iii) adult female (hybridization with sense probe, background control). (iv–vii) enlarged views of (iv) epithelium, (v) ovaries, and (vi and vii) digestive cells of adult female, taken from independently stained mites. G–I, Effects of dsRNA-*Tu-CPR*. G, Relative level of *Tu-CPR* transcript normalized with *RP49* in dsRNA-treated Col-a mites (mean ± sem, *n* = 8). H, The activity of cytochrome P450 in dsRNA-treated Col-a mites upon feeding on Col-0 plants (mean ± sem, *n* = 5). I, Fecundity of dsRNA-treated Col-a mites feeding on Col-0 or bean plants. Fecundity was measured over 2 d (third and fourth dpi) and data are presented as mean number of eggs laid by a female mite per day ± sem, *n* = 30 for Col-0 plants and *n* = 12 for bean plants. See also Supplemental Figure S5. Asterisks indicate a significant difference between treated and control samples (unpaired Student’s *t* test: *P < 0.05, **P < 0.01, ***P < 0.001). Scale bars in (F): (i–iii) 20 μm; (iv–vii) 100 μm. Individual sample values are shown as open circles for *n* ≤ 10.

To test the requirement of esterase, GST, and P450 activities for TSSM adaptation to Arabidopsis, we used S,S,S tributyl-phosphorotrithioate (DEF; an inhibitor of esterase activity), diethyl maleate (DEM; an inhibitor of GST activity), and piperonyl butoxide (PBO) and trichlorophenylpropynyl ether (TCPPE; inhibitors of P450 activity) to reduce the activity of the indicated enzymatic classes. If a particular class of enzymes is required for mite adaptation to Arabidopsis, then the inhibition of its activity is expected to restore the susceptibility of Arabidopsis-adapted mites to Arabidopsis defense compounds. Concentrations of 2,000 mg/L DEM, 100 mg/L DEF, 1,000 mg/L PBO, and 1,500 mg/L TCPPE were used as they cause <10% mite mortality (Supplemental Figure S3), but were nevertheless capable of significantly reducing the corresponding enzymatic activities in Col-a mites feeding on Col-0 plants (Figure 5, A and C; Supplemental Figure S4). As inhibitors do not affect all enzymes equally within the targeted enzymatic class (Feyereisen, 2014), the lack of inhibitory effect on mite performance is not strong evidence against the involvement of a particular enzymatic class in mite host adaptation. Conversely, the significant decrease of mite performance on a new host plant upon the application of a given inhibitor strongly supports the requirement of enzyme(s) within the corresponding class for mite host adaptation.

The application of DEF reduced esterase activities by 32% but did not affect the fitness of Col-a mites when feeding on either Col-0 or *cyp79b2 cyp79b3* plants (Supplemental Figure S4), indicating that esterases may not be required for mite adaptation to Arabidopsis. The application of DEM decreased the GST enzymatic activity by 43% and had a minor but significant effect on the fecundity of Col-a mites (Supplemental Figure S4), suggesting that GST activity is required for high Col-a performance on Arabidopsis.

The application of PBO significantly reduced the P450 activity (25%) and dramatically reduced (44%) the fecundity of Col-a mites feeding on Col-a host plants, and to a lesser extent when they fed on *cyp79b2 cyp79b3* plants (Figure 5, A and B). Since PBO does not interfere with all P450s equally and also inhibits esterases in some arthropods (Feyereisen, 2014), we applied another structurally unrelated P450 inhibitor, TCPPE, to confirm that the observed effects were due to the decreased P450 activity. TCPPE significantly inhibited P450 enzyme activity in Col-a mites (13%) and dramatically reduced the fecundity of Col-a mites that fed on Col-0 (39%) and *cyp79b2 cyp79b3* plants (33%), (Figure 5, C and D). In addition, the fitness of bean-a mites decreased by 13% when they fed on *cyp79b2 cyp79b3* TCPPE-treated plants (Figure 5D).

### P450 activity is required for TSSM adaptation to Arabidopsis

As P450 inhibitors were applied directly to Arabidopsis leaves, there is a possibility that PBO/TCPPE perturbed Arabidopsis defense physiology and only secondarily affected mite fecundity. For example, the synthesis of JA metabolites (Heitz et al., 2012; Aubert et al., 2015; Widemann et al., 2015) and Trp-derived secondary metabolites (Hull et al., 2000) occur via pathways that include many plant P450s. Therefore, to confirm the requirement of mite P450s in TSSM adaptation to Arabidopsis, we have used a recently developed RNAi gene silencing protocol in TSSM (Suzuki et al., 2017; Bensoussan et al., 2020) to silence the P450 pathway in mites.

Because the *CYP* (PF00067) gene family has over 100 *CYP* genes (Grbic et al., 2011; Mistry et al., 2020), it is difficult to characterize the involvement of individual P450s in mite adaptation to Arabidopsis using RNAi. To circumvent this hurdle, we instead silenced the expression of *NADPH-cytochrome P450 reductase* (*Tu-CPR*), a co-enzyme required for the catalytic reactions carried out by microsomal P450s (Masters and Okita, 1980). *Tu-CPR* (*tetur18g03390)* is a single copy gene (Grbic et al., 2011) constitutively expressed in all tissues of adult spider mites, including the midgut epithelial and digestive cells (Figure 5F) where digestion and detoxification of dietary xenobiotics are expected to take place (Bensoussan et al., 2018). Application of dsRNA-*Tu-CPR* to Col-a mites resulted in decreased expression of *Tu*-*CPR* (Figure 5G). Consistent with the Tu-CPR function as an essential component of the P450 enzyme complex, P450 activity was reduced by 38% in the *Tu-CPR* silenced mites relative to mites exposed to dsRNA-NC (Figure 5H). Strikingly, the silencing of *Tu-CPR* reduced the fecundity of Col-a mites by almost 50% when they fed on Col-0 plants (Figure 5I). Similar phenotypes were obtained upon the application of a second, independent, dsRNA-*Tu-CPR-1* fragment (Supplemental Figure S5), confirming the specificity of the observed phenotypes to a loss of *Tu-CPR* function and the requirement of P450 activity for the adaptation of Col-a mites to Arabidopsis. The reduced P450 activity, through the application of P450 inhibitors or dsRNA-*Tu-CPR*, did not affect TSSM fecundity when mites fed on bean plants (Figure 5, E and I; Supplemental Figure S5), demonstrating that P450 activity is specifically required for mites’ ability to use Arabidopsis as a host.

## Discussion

The innate ability to perceive and respond to plant defenses in a process of physiological acclimation is assumed to enable broad-generalist herbivores to utilize a wide range of host plants. On the other hand, genetic adaptation is presumed to enable specialist herbivores to evolve novel adaptation traits that efficiently counteract the defenses of a narrow range of host plants (Despres et al., 2007; Barrett and Heil, 2012; Heidel-Fischer and Vogel, 2015). However, our understanding of the host-shift responses in composite generalist herbivores that exist as complexes of host-specialized populations is not well understood. TSSM is an example of a composite generalist herbivore that feeds on over a thousand plant hosts. Individual mite populations thrive on a subset of potential hosts but can rapidly adapt to new plant hosts (Gould, 1979; Fry, 1989; Agrawal et al., 2002; Magalhaes et al., 2007; Fellous et al., 2014; Wybouw et al., 2015). Thus, mite host adaptability is one of the key features underlying the extremely wide host range of TSSM.

TSSM, like other polyphagous herbivores, acclimates to a host shift by reprogramming its salivary and detoxification complements (Figure 4; Dermauw et al., 2013; Zhurov et al., 2014; Wybouw et al., 2015; Villarroel et al., 2016; Jonckheere et al., 2018, 2016). However, exposure to initially unfavorable host plants over several generations enables TSSM to dramatically increase its performance (Figure 2; Gould, 1979; Fry, 1989; Magalhaes et al., 2007; Wybouw et al., 2015). Here, we used Arabidopsis and bean-a mites to investigate mechanisms that mediate the formation of host-adapted populations in this generalist herbivore. Arabidopsis is a nonpreferred host for the bean-a mite population (Figure 1). However, under experimental selection, bean-a mites gave rise to Arabidopsis-adapted *cyp*-a and Col-a populations (Figure 2). Matching the complexity of Arabidopsis defenses, Arabidopsis-adapted populations evolved adaptation traits capable of counteracting *CYP79B2 CYP79B3*-independent Arabidopsis defenses (present in both *cyp*-a and Col-a mites) and Trp-derived defenses (present only in Col-a mites). Remarkably, these complex adaptation traits were established within 25 generations of mite Arabidopsis selection.

Suppression of plant defenses has been proposed to be one of the mechanisms of mite host adaptation (Villarroel et al., 2016; Blaazer et al., 2018; Jonckheere et al., 2018, 2016). Attenuation of host-induced responses has been described for several spider mite species in their interactions with the tomato (*Solanum lycopersicum*) plant host (Kant et al., 2008; Glas et al., 2014; Sarmento et al., 2011; Godinho et al., 2016). The prevalence of the ability to suppress plant responses across tomato-adapted TSSM populations is currently not known, nor is its presence in other TSSM host-adapted populations. Even though the interference with either JA-biosynthesis or its signaling could have been an effective way to attenuate a whole range of Arabidopsis defense compounds (Figure 1; Zhurov et al., 2014; Widemann et al., 2021), our data suggest that Arabidopsis-adapted mites did not interfere with JA-regulated Arabidopsis defenses. Arabidopsis-adapted mites, just like the ancestral bean-a population, had greater fitness on *cyp79b2 cyp79b3* than on Col-0 plants (Figure 2). They also induced the expression of JA-responsive genes and accumulated I3M to levels similar to those induced by the bean-a mites (Figure 3). This is surprising, as the ancestral bean-a strain used in this study previously gave rise to tomato-adapted populations that, in the process, gained the ability to suppress tomato mite-induced responses (Wybouw et al., 2015), raising the question if and how host defenses affect the mechanism of TSSM adaptation. Instead, we provided multiple independent lines of evidence indicating that Arabidopsis-adapted mites evolved metabolic resistances to counteract Arabidopsis defenses.

The pharmacological experimental treatments identified the requirement of GSTs for the fitness advantage of Col-a over bean-a mites when they fed on Col-0 plants (Supplemental Figure S4). GST-based detoxification has been associated with the ability of numerous generalist herbivores to counteract the anti-herbivore effects of glucosinolates, considered to be the major class of Arabidopsis defense compounds (Wadleigh and Yu, 1988; Schramm et al., 2012; Gloss et al., 2014; Jeschke et al., 2016). Aliphatic and indole glucosinolates are the most abundant classes of these compounds in Arabidopsis (Brown et al., 2003). Herbivores have differential susceptibility to aliphatic and indole glucosinolates, so that some are exclusively affected by the aliphatic (Müller et al., 2010), some by the indole (Kim and Jander, 2007), and some by both classes of glucosinolates (Müller et al., 2010; Jeschke et al., 2017). Aliphatic glucosinolates are ineffective in restricting mite herbivory (Zhurov et al., 2014). Instead, the antifeedant effects of indole glucosinolates on TSSM have been recently characterized (Widemann et al., 2021), raising a possibility that Arabidopsis defensive indole glucosinolate breakdown products may be detoxified by mite adaptation-associated GST(s).

The pharmacological and RNAi reverse genetics experiments pointed to the critical requirement of P450 activities for the adaptation of Col-a mites to Arabidopsis (Figure 5; Supplemental Figure S4). We identified two distinct modes of P450 activities. One corresponds to the initial mite response to the shift from bean to Arabidopsis that is similar in both ancestral and Arabidopsis-adapted mites (Figure 4). The inhibition of these P450s had a limited but significant effect on the fecundity of the bean-a ancestral TSSM population when these mites were challenged on the Arabidopsis host (Figure 5D). As bean-a mites had no prior exposure to Arabidopsis plants, the effect of P450 inhibitors on their fecundity uncovers the limited contribution of initially induced P450s to mite fitness on Arabidopsis*.* Since Arabidopsis-adapted mites retained responsiveness to shift from bean to Arabidopsis plants (Figure 4), it is expected that these P450s enable limited mite adaptation to Arabidopsis defenses (Zhurov et al., 2014; Figure 5). This is consistent with the physiological acclimation described for broad generalists whereby exposure to plant xenobiotics is assumed to result in the transcriptional induction of genes encoding enzymes that recognize a wide range of substrates and have low enzymatic catalytic activity (Li et al., 2004; Sasabe et al., 2004; Wen et al., 2006; Halon et al., 2015; Mathers et al., 2017; Shi et al., 2018; Wang et al., 2018; Snoeck et al., 2019). The other more substantial contribution of P450-mediated metabolic resistance is realized through the action of highly effective P450 detoxification responses against Arabidopsis defenses that are exclusively associated with mite adaptation to Arabidopsis. The reduction of P450 activity in Col-a mites diminished their fitness advantage over ancestral bean-a mites, demonstrating that adaptation-associated P450 activity is the main contributor to mite resistance to Arabidopsis defenses (Figure 5, B, D, and I; Supplemental Figure S5). The inhibition of P450 activity reduced Col-a mite fecundity when they fed on both Col-0 and *cyp79b2 cyp79b3* plants (Figure 5, B and D), suggesting that adaptation-associated P450s mainly counteract CYP79B2 CYP79B3-independent Arabidopsis defenses whose identity is currently not known. However, the requirement of P450 activity for the adaptation of green peach aphid (*Myzus persicae*) to Arabidopsis indole glucosinolates (Ji et al., 2021) raises the possibility that mite adaptation-associated P450s may also counteract the toxicity of indole glucosinolate-associated defensive compounds.

In summary, as demonstrated here for the Arabidopsis-adapted mite populations, TSSM can evolve resistance against complex plant defenses within a mere 25 generations. Arabidopsis-adapted mites retained the ability to acclimate to the shift from bean to Arabidopsis. Such transcriptional plasticity, characteristic of broad generalists, is shared between ancestral and Arabidopsis-adapted mites and has a small contribution to mite fitness on Arabidopsis. At present, it is not clear if these initial responses are common stress/xenobiotic responses to any novel host, nor if they are required for mite host adaptation. If they are, they may provide initial fitness benefits upon mites’ shift to a new host that may facilitate the evolution of the host adaptation traits. We further identified the highly effective P450 detoxification responses that are required for mite adaptation to Arabidopsis. This pattern of metabolic counteraction is reminiscent of strategies deployed by host-specialized herbivores, in which host adaptation is associated with either nucleotide substitutions that enhance the enzyme activity (Li et al., 2007; Schuler, 2011) or with the overexpression of detoxification genes (Bass et al., 2013). The *CYP* gene family underwent a lineage-specific expansion in TSSM and consists of 115 genes in bean-a mites (Grbic et al., 2011; Mistry et al., 2020). Intraspecific genetic variability is expected at *CYP* loci as some appear as clusters of duplicated genes that are prone to the formation of chimeric genes or copy number variation. Comparative genome and transcriptome analysis of Arabidopsis-adapted and ancestral TSSM populations may identify candidate adaptation-required *CYP* gene(s). In humans, for example, only 15 out of 57 P450s are involved in xenobiotic metabolism (Handschin and Meyer, 2003). The comparative sequence and functional analysis of candidate *CYPs* will reveal if adaptation-required P450 activity is achieved by altering gene regulation, copy number, or through substitution(s) that increase enzyme activity and if these changes resulted from the selection within the genetic pool of the founding population, through de novo mutations or epigenetic changes.

## Materials and methods

### Plant growing and mite rearing conditions

Seeds of *A.* *thaliana* Col-0 were obtained from the Arabidopsis Biological Resource Center (Ohio State University), *cyp79b2 cyp79b3* (Zhao et al., 2002) from B.A. Halkier (University of Copenhagen, Denmark), *myc2 myc3 myc4* (Fernandez-Calvo et al., 2011) from R. Solano (Universidad Autónoma de Madrid), and bean (*P.* *vulgaris*, cultivar California Red Kidney) from Stokes, Thorold, Ontario, Canada. Plants were grown under 100–150 μmol m^−2^ s^−1^ cool-white fluorescent light at 24°C with a 10-h/14-h (light/dark) photoperiod in controlled growth chambers. TSSMs (*T.* *urticae*), London reference strain (Grbic et al., 2011), were reared on bean plants at 24°C, 60% relative humidity, and with a 16-h/8-h (light/dark) photoperiod for >10 years.

### Experimental evolution

Approximately 1,000 randomly chosen adult bean-a female mites were transferred to 3-week-old *cyp79b2 cyp79b3* or Col-0 Arabidopsis plants that were replaced biweekly. Mite populations were allowed to propagate for 18 months, generating six selected lines: #1–#3 independent lines each selected on *cyp79b2 cyp79b3* and Col-0 plants, referred to as *cyp*-a and Col-a lines, respectively. Selected lines were reared on their corresponding plant hosts under the same conditions as the ancestral strain. *cyp*-a (#3) and Col-a (#3) were used for the follow-up experiments.

### Mite performance analysis

Twenty 3-d-old adult female mites were transferred to each of the experimental plants (bean, *cyp79b2 cyp79b3*, and Col-0), and total population size at 7-d postinoculation (dpi) was evaluated in direct (mites were moved directly from their rearing hosts to experimental plants) and indirect (mites were reared on bean plants for two generations before their transfer to the experimental plants) transfer regimes. The experiment was performed in four biological replicates for each selected line. Differences between selected line population sizes were determined using a one-way ANOVA using mite strain as the main effect, followed by Tukey’s HSD test. All other experiments were performed with mites that were propagated for two generations on bean plants, thus, in the indirect transfer regime.

### Plant damage analysis

Leaf damage of *cyp79b2 cyp79b3* and Col-0 Arabidopsis plants upon feeding of bean-a, *cyp*-a, and Col-a mite strains was performed as previously described (Zhurov et al., 2014). Briefly, 10 adult female mites were placed on the rosette of Arabidopsis plants and allowed to feed for 3 d before plants were cut at the base of the rosette. The adaxial side of the rosette was scanned using a Canon CanoScan 8600F model scanner (Canon USA Inc., Melville, NY, USA) at a resolution of 1,200 dpi and a brightness setting of +25. Scanned plants were saved as .jpg files for subsequent analysis in Adobe Photoshop 5 (Adobe Systems, San Jose, CA, USA). The experiment was performed using six biological replicates/trial, and in three experimental trials, *n* = 18. Differences in plant damage between mite strains on different hosts were determined using a three-way ANOVA, using mite strain, plant host, and experimental trial as main effects, including interaction terms, followed by Tukey’s HSD test. The inclusion of experimental trial and its potential interactions with main effects was used both to control for the effect of trial and to test for reproducibility across trials, as suggested by (Brady et al., 2015). This statistical approach was used in all following analyses.

### Mite fecundity assay

Fecundity over 2 d of bean-a mites on Col-0, *cyp79b2 cyp79b3, and myc2 myc3 myc4* plants, and fecundity over 6 d of bean-a, *cyp*-a, and Col-a mites on *cyp79b2 cyp79b3* or Col-0 Arabidopsis plants were assessed as previously described (Widemann et al., 2021). The experiment included 5–10 biological replicates for each treatment that were repeated in three experimental trials. A two- and three-way ANOVA was performed respectively, using the mite strain, plant genotype, and experimental trial as main effects including relevant two-way interaction terms when required. For the mite fecundity assay on MeJA-treated Col-0 leaves, the leaves from 5-week-old Col-0 plants were sprayed 6 times over 48 h with MeJA (Sigma-Aldrich, St Louis, MO, USA; Cat # 392707, 500 µM in ethanol 0.4% (v/v)), as previously described (Feldberg et al., 2009). On the second day of treatment, fully elongated leaves were detached and a day later infested with 10 adult female bean-a mites whose fecundity was recorded after 24 h. The experiment included nine biological replicates and was repeated in two experimental trials. Differences in fecundity were detected by a two-way ANOVA, using the treatment and experimental trial as main effects including an interaction term.

### RT-qPCR and metabolic analyses

For plant marker gene and metabolite analyses, 10 3-d-old adult female bean-a, *cyp*-a, and Col-a mites, propagated for two generations on bean plants, were allowed to feed on Col-0 plants for 24 h after which whole rosettes were collected. For TSSM marker gene analysis, samples of 100 3-d-old adult female bean-a, *cyp*-a, and Col-a mites, initially propagated for two generations on bean plants and then transferred to bean, *cyp79b2 cyp79b3*, or Col-0 plants for 24 h, were collected. Both experiments were replicated in three biological replicates/trial and three independent trials. Preparation of RNA, cDNA, and the RT-qPCR analysis was performed as previously described (Zhurov et al., 2014). Primer sequences and amplification efficiencies used in qPCR are shown in Supplemental Table S1. *PEROXIN4* (*AT5G25760*) and *RIBOSOMAL PROTEIN 49* (*tetur18g03590*) were used as the reference genes for Arabidopsis and mite genes, respectively. Ct values of three technical replicates were averaged to generate a biological replicate Ct value. Plotting and statistical analysis were performed as described previously (Rieu and Powers, 2009). The quantification of plant metabolites (JA, JA-Ile, and I3M) was performed as described in Zhurov et al., 2014. Briefly, jasmonates (JA and JA-Ile) were analyzed in aqueous extracts by mass spectrometry monitoring their specific precursor-to-product ion transition in a triple quadrupole mass spectrometer (Micromass Ltd. Wilmslow, UK). Quantitation was achieved after external calibration with standards of known amount considering the analyte/internal standard ratio (dihydrojasmonic acid, added before extraction). Moreover, I3M was identified and analyzed following a nontargeted approach using a hybrid quadrupole-time-of-flight mass spectrometer. Identification of I3M was performed after inspection of its specific mass spectrum in negative electrospray ionization mode: 447.053 [M-H]^−^, 253.03 [C_10_H_9_N_2_O_4_S]^−^, 96.95 [HSO_4_]^−^. Relative quantitation of I3M was attained by dividing peak areas of I3M and internal standard (biochanin A, 283.07 [M-H]^−^). The resulting value was again divided by the actual sample weight expressed in milligram. Mite marker gene data were analyzed by a two-way ANOVA with mite strain and plant host as main effects including an interaction term, followed by Tukey’s HSD test. For plant marker gene and metabolite analysis, the data were analyzed by a two-way ANOVA, using mite strain and experimental trial as main effects including an interaction term, followed by Tukey’s HSD test. For a graphical representation of the expression of plant marker genes, the NRQ data were further normalized to the “no mite” control sample, which was therefore set to one. Thus, these results represent fold change differences in the expression of marker genes relative to “no mite” control.

### Determination of cytochrome P450, esterase, and GST activity

To perform enzymatic activity assays, 3-d-old spider mite females of each Col-a, *cyp*-a, and bean-a strain, reared for two generations on bean plants, were placed on bean, *cyp79b2 cyp79b3*, or Col-0 plants. After 24 h, 200 females from each treatment were collected and were used for protein extraction and enzymatic assays. Mites were homogenized in 700 µL of 100 mM phosphate buffer, pH 7.6. The concentration of total protein mite extracts was determined using the Quick Start Bradford Protein Assay (Quick start Bradford 1× dye reagent, Bio-Rad, Hercules, CA, USA; Cat# 500-0205), with Bovine Serum Albumin (Sigma-Aldrich; Cat# A7906) as the standard. Twenty, 10, and 10 µg of protein per reaction were used for measuring the enzymatic activity of GSTs, P450s, and esterases, respectively. The enzymatic activities were assessed spectrophotometrically using 1-chloro-2,4-dinitrobenzene (for GSTs; Habig and Jakoby, 1981), 7-ethoxy-4-trifluoromethylcoumarin (for P450s) (Buters et al., 1993), and 4-nitrophenyl acetate (for esterases; Park et al., 1961), as substrates. For GST and esterases, the linearity of the reaction was determined by plotting the absorbance values against time over a 5 min period and calculation of sample activity was performed in the linear range. All assays were corrected for the occurrence of nonenzymatic substrate transformation in blank samples in which the protein solution was replaced by the buffer. Enzymatic activities were determined in four independent samples with three technical replicates per sample. Differences in enzymatic activity between mite strains on different plant hosts were detected using a two-way ANOVA, with mite strain and plant host as main effects including an interaction term, followed by Tukey’s HSD test.

### Application of enzyme inhibitors

Solutions of 2,000 mg/L DEM (an inhibitor of GST activity), 100 mg/L DEF (an inhibitor of esterase activity), 1,000 mg/L PBO, and 1,500 mg/L TCPPE (inhibitors of P450 activities) were used as they cause <10% mortality of bean-a mites (Supplemental Figure S3). Detached leaves of Col-0 Arabidopsis plants were dipped in one of the DEF, DEM, PBO, or TCPPE solutions or water and acetone 0.1% (v/v) as the control. Upon drying, leaves were infested with either one adult bean-a or Col-a female mite and the number of eggs laid by each female per day was recorded for 6 d, or with 10 2-d-old adult Col-a female mites that were collected after 24 h. A pool of 100 spider mites was used to measure GST, esterase, and P450 activities in DEM-, DEF-, PBO-, and TCPPE-treated spider mites, respectively. Fecundity was determined in three independent experimental trials with 10 replicates per trial. Enzymatic activities were determined in five independent samples with three technical replicates per sample. Differences between means of fecundity and enzymatic activities of control and inhibitor-treated samples were calculated using unpaired Student’s *t* tests.

### In situ hybridization

DIG-labeled probes were produced and the whole-mount in situ hybridization was performed according to previously published methods (Dearden et al., 2002). Images were collected using a Zeiss AxioCam HRc 412-312 camera mounted on a Zeiss Axioplan II microscope.

### RNAi of *Tu-CPR*

Two nonoverlapping fragments, *Tu-CPR* (645 nt) and *Tu-CPR-F1* (564 nt), complementary to the coding region of *Tu-CPR (tetur18g03390*) and dsRNA complementary to a 382 bp nontranscribed intergenic fragment (1690614–1690995 of the genomic scaffold 12) used as a negative control dsRNA (referred to as NC) were synthesized using primers listed in Supplementary Table S1 as described in (Suzuki et al., 2017). A BLAST search against the *T. urticae* genome confirmed that dsRNA sequences are unique. dsRNA solutions at concentrations of 500 ng/µL were supplemented with 6% (v/v) blue dye erioglaucine (McCormick, Sparks Glencoe, MD, USA). Newly molted adult female mites were soaked in dsRNA/dye solutions at 20°C for 24 h (Suzuki et al., 2017). Postsoaking, mites with visible blue dye in the posterior midgut were selected and were transferred in batches of 10 to either detached Col-0 leaves or bean leaf disks. Fecundity was determined in three independent experimental trials with 10 replicates/trial as a number of eggs deposited by individual female mite on the third and fourth-day postsoaking. For the analysis of *CPR* expression, 100 adult female mites were treated with either dsRNA-*Tu-CPR*, dsRNA-*Tu-CPR-1*, or dsRNA-NC. Mites were allowed to feed on Col-0 leaves for 4 d. Thereafter, mites were selected based on visual phenotype (spotless) and were used for the analysis of *CPR* expression levels. The RT-qPCR was performed in eight (for dsRNA-*CPR*) and three (for dsRNA-*CPR-1*) experimental replicates, as described above. For the analysis of P450 activity in *CPR* silenced mites, 100 adult female mites were treated with either dsRNA-*Tu-CPR*, dsRNA-*Tu-CPR-1*, or dsRNA-NC. After 4 d of feeding on Col-0 leaves, mites were collected and the P450 activity was determined in five independent samples with three technical replicates per sample, as described above. Differences in fecundity between dsRNA-treated mites were detected using a two-way ANOVA, with mite treatment and experimental trial as main effects including an interaction term, followed by Tukey’s HSD test. Enzyme activity and RT-qPCR expression levels were compared between dsRNA-treated mites using unpaired Student’s *t* tests. Independent mite samples were used for the fecundity assay and the analysis of transcript levels and P450 activity.

### Accession numbers

Sequence data from this article can be found in the GenBank/EMBL data libraries under accession numbers *PEROXIN4, AT5G25760; RIBOSOMAL PROTEIN 49, tetur18g03590; Tu-CPR, tetur18g03390*.

## Supplemental data 

The following materials are available in the online version of this article.

**Supplemental Figure S1.** Experimental evolution of TSSM adaptation to Arabidopsis.

**Supplemental Figure S2.** The cytochrome P450, GST and esterase activities in bean-a, *cyp*-a, and Col-a mites (reared on beans for two generations) feeding on bean, *cyp79b2 cyp79b3*, or Col-0 plants.

**Supplemental Figure S3.** Selection of experimental concentrations of enzyme inhibitors.

**Supplemental Figure S4.** The requirements of esterase and GST activities for TSSM adaptation to Arabidopsis.

**Supplemental Figure S5.** RNAi silencing of *Tu-CPR*.

**Supplemental Table S1.** Gene-specific primer sequences used for RT-qPCR.

## Supplementary Material

kiab412_Supplementary_Data

## Notes

Investigation: G.S., K.B., H.R., V.A., S.D., E.W., M.M., and V.Z.; writing: G.S., K.B., V.Z., M.G., and V.G.; supervision: V.Z., A.G.C., M.G., and V.G.; conceptualization, funding acquisition, and resources: A.G.C., M.G., and V.G.

The author responsible for distribution of materials integral to the findings presented in this article in accordance with the policy described in the Instructions for Authors (https://academic.oup.com/plphys/pages/General-Instructions) is Vojislava Grbic (vgrbic@uwo.ca).
