# Supplementary material for: Rapid specialization of counter defenses enables two-spotted spider mite to adapt to novel plant hosts
Source: Plant Physiol. 2021 Aug 31;187(4):2608–22. doi: 10.1093/plphys/kiab412 (PMC8644343; doi:10.1093/plphys/kiab412)
Supplement: kiab412_Supplementary_Data [file kiab412_supplementary_data.pdf]

Supplemental Table and Figures Legends

Supplemental Figure S1. Experimental evolution of TSSM adaptation to Arabidopsis. Performance of ancestral and selected populations on bean (top), *cyp79b2 cyp79b3* (middle) and Col-0 (bottom) plants. Mites were transferred directly from their respective rearing hosts to experimental bean, *cyp79b2 cyp79b3* and Col-0 plants. The performance was measured as the size of the total population derived from twenty adult female mites and seven days post infestation. Data are represented as mean ± SEM, *n* = 4. Statistics were performed on total population counts. Different letters represent significant differences between means (Tukey's HSD test, α = 0.05). Individual sample values are shown as open circles for n ≤ 10.

Supplemental Figure S2. The activities of cytochrome P450 (**A**), glutathione-S-transferases (**B**) and esterase (**C**) in bean-a, *cyp*-a and Col-a mites (reared on beans for two generations) feeding on bean, *cyp79b2 cyp79b3* or Col-0 plants. Data are represented as mean ± SEM, *n* = 4. Different letters represent significant differences between means (Tukey's HSD test, α = 0.05), following three-way ANOVA. The plant host - mite strain interaction was significant for the activity of cytochrome P450 (F = 103.93, P = 5.451e-16). Individual sample values are shown as open circles for n ≤ 10.

Supplemental Figure S3. Selection of experimental concentration of enzyme inhibitors. Data are presented as mean mortality of a female mite at 3 dpi ± SEM (for treatment and control *n* = 10 for PBO, DEM and DEF experiments, and *n* = 9 for TCPPE experiment). Individual sample values are shown as open circles for n ≤ 10.

Supplemental Figure S4. The requirements of esterase and glutathione-S-transferase (GST) activities for TSSM adaptation to Arabidopsis. The GST (**A**) and esterase (**C**) activities in Col-a mites feeding on Col-0 plants after the application of diethyl maleate (DEM, an inhibitor of GST activity, in **A**) and S,S,S tributyl-phosphorotrithioate (DEF, an inhibitor of esterase activity, in **C**). Data are represented as mean ± SEM, *n* = 5. **B** and **D**, Effects of DEM (in **B**) and DEF (in **D**)

treatments on fecundity of bean-a and Col-a mites upon feeding on Col-0 and *cyp79b2 cyp79b3* plants. Fecundity was assessed as mean number of eggs laid by a female mite in six days ± SEM, n = 30. Asterisks in panels **A**-**C** indicate a significant difference between treated and control samples (unpaired Student's t test: *P < 0.05, **P < 0.01). Individual sample values are shown as open circles for n ≤ 10.

Supplemental Figure S5. RNAi silencing of *Tu-CPR*. **A**, A schematic of the *Tu-CPR* locus. DNA sequences used for the generation of dsRNA-*Tu-CPR* are shown in red (fragment CPR, 645 bp), and green (fragment CPR-1, 564 bp). UTR and coding sequences are shown as light and dark blue boxes, respectively. **B**, The effect of dsRNA-*Tu-CPR*. Relative expression of *Tu-CPR* normalized with *RP49* in dsRNA treated Col-a mites (mean ± SEM, n = 8, Student's t test, ns). **C**, Fecundity of dsRNA treated Col-a mites feeding on Col-0 and bean plants. Fecundity was measured over two days (3 and 4 dpi) and data are presented as mean number of eggs laid by a female mite per day ± SEM, n = 30 for Col-0 plants and n = 12 for bean plants (Student's t test *** P < 0.001). Individual sample values are shown as open circles for n ≤ 10.

Supplemental Table S1. Gene-specific primer sequences used for RT-qPCR.

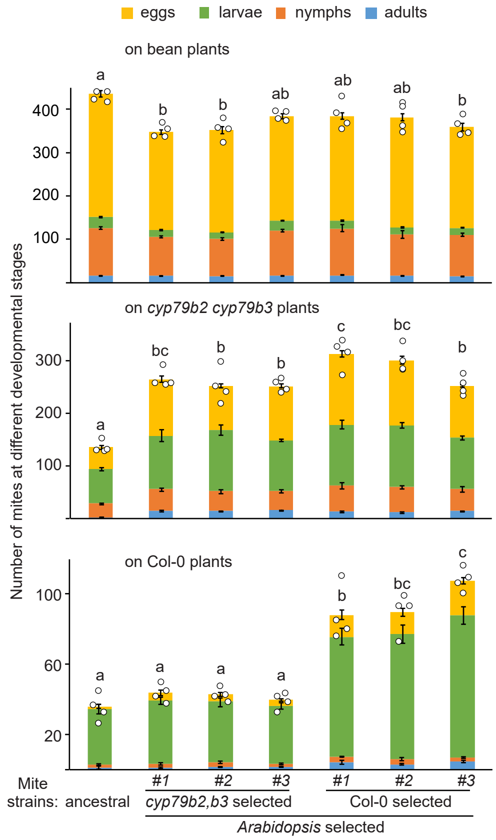

Supplemental Figure S1

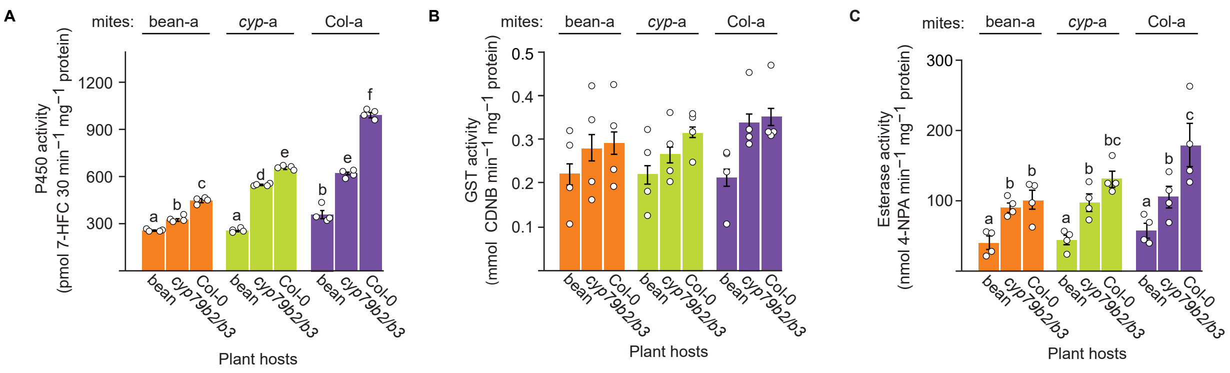

Supplemental Figure S2

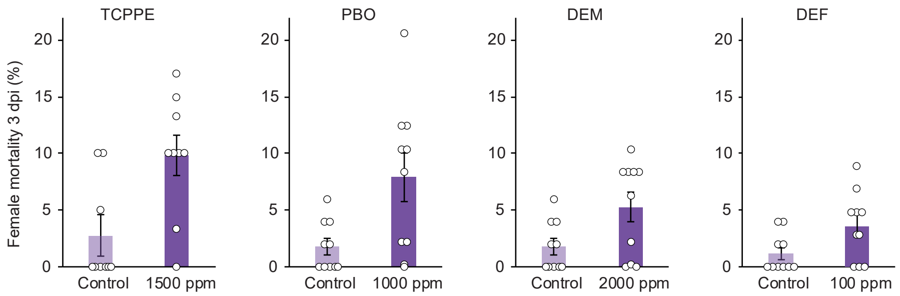

Supplemental Figure S3

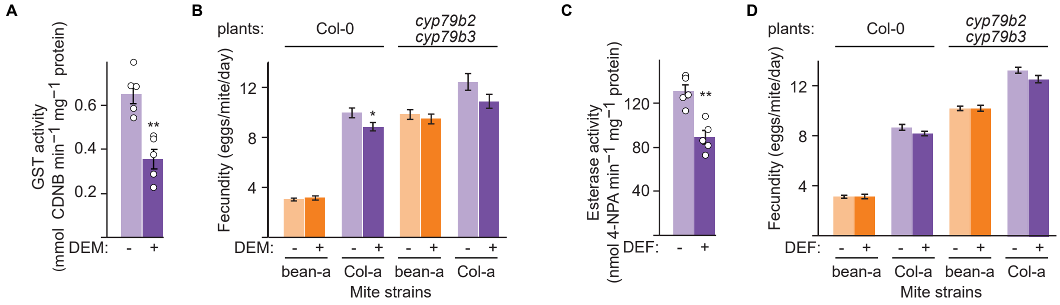

Supplemental Figure S4

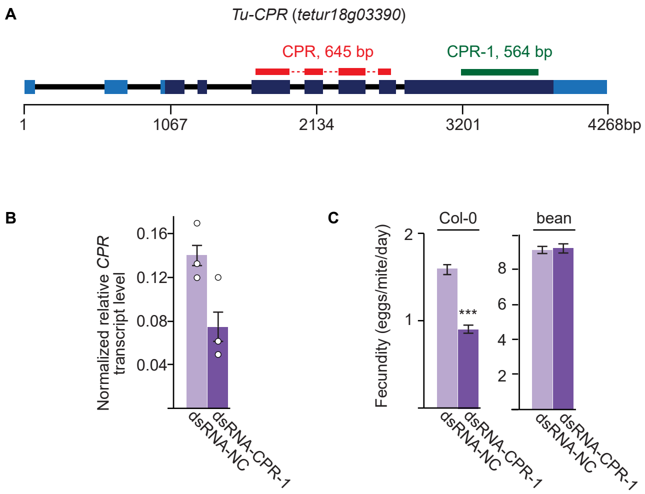

Supplemental Figure S5

Supplemental Table S1.

Locus ID and name	Application	Primers	Efficiency
tetur07g06410 *CYP392A1*	RT-qPCR	F: 5'-GACGCCTTCGCAAATGATGC-3'<br>R: 5'-TAACCGTCTGTGTTCACGCC-3'	0.929
tetur06g04520 *CYP392A16*	RT-qPCR	F: 5'-TTGATTGGGCTTGCCCTCTT-3'<br>R: 5'-AGCCAACAATCGGAAGACCC-3'	0.903
tetur03g05070 *CYP392D8*	RT-qPCR	F: 5'-ACCAGAGAGATTCCTCAGCG-3'<br>R: 5'-AAAGCCAAAGTTGCACCAGG-3'	1.030
tetur02g02480 *UGT201A2v2*	RT-qPCR	F: 5'-TCGAGAACTGAGTGGAGTAGC-3'<br>R: 5'-TAGCAGGCAAAGGTGTTCCA-3'	1.090
tetur02g09830 *UGT204B1*	RT-qPCR	F: 5'-GCTTCGGTTGAGAAACGTGG-3'<br>R: 5'-AAAATCGGCATTCGCTTCGG-3'	1.000
tetur05g00090 *UGT204A5*	RT-qPCR	F: 5'-TGGACGGAAATCGTAGTGGA-3'<br>R: 5'-AGCTCATCAAAGACCAGCGA-3'	1.090
tetur18g03590 *RP49*	RT-qPCR	F: 5'-CTTCAAGCGGCATCAGAGC-3'<br>R: 5'-CGCATCTGACCCTTGAACTTC-3'	0.976
AT5G42650 *AOS*	RT-qPCR	F: 5'-AAATCCAACGGCGGAGAACT-3'<br>R: 5'-TCGTCGCCAACGGTTGATAA-3'	0.984
AT4G39950 *CYP79B2*	RT-qPCR	F: 5'-GAAAAGAGGTTGTGCGGCTC-3'<br>R: 5'-TCTCACTTCACCGTCGGGTA-3'	0.994
AT2G22330 *CYP79B3*	RT-qPCR	F: 5'-TCTACCGATGCTTACGGGATTG-3'<br>R: 5'-TACAAGTTCCTTAATGGTTGGTTTG-3'	0.973
AT1G32640 *MYC2*	RT-qPCR	F: 5'-TCGCTTACATCAACGAGCTTAAATC-3'<br>F: 5'- TATCTTCACTTCAATCTCCATCCCC-3'	0.900
AT5G25760 *PEROXIN4*	RT-qPCR	F: 5'-GCTCTTATCAAAGGACCTTCGG-3'<br>R: 5'-CGAACTTGAGGAGGTTGCAAAG-3'	0.992
tetur18g03390 *Tu-CPR*	RT-qPCR	F: 5'-CCATTCTTGGCACCTATCGT-3'<br>R: 5'-GCAAGGTGATCTCCAGCTTC-3'	0.994
tetur18g03390 *Tu-CPR*	RNAi (fragment 1)	F: 5'-[T7]-CCTCGACTTCAGCCACGTTA-3'<br>R: 5'-[T7]-AACATCCCGAGCCATGTTCC-3'	not applicable
tetur18g03390 *Tu-CPR*	RNAi (fragment 2)	F: 5'-[T7]-ACAAACCGGTACTGCAGAGG-3'<br>R: 5'-[T7]-TGCATACACGAACGGTCTCC-3'	not applicable
T. urticae genomic scaffold 12, position 1690614-1690995, *NC*	RNAi (negative control)	F: 5'-GCCCTCTCCTGGTTGTAAACTT-3'<br>R: 5'-CGACCCCATCAGGCTATTGA-3'	not applicable
tetur18g03390 *Tu-CPR*	*in situ* (antisense)	F: 5'-[T7]-ACAAACCGGTACTGCAGAGG-3'<br>R: 5'-TGCATACACGAACGGTCTCC-3'	not applicable
tetur18g03390 *Tu-CPR*	*in situ* (sense)	F: 5'-ACAAACCGGTACTGCAGAGG-3'<br>R: 5'-[T7]-TGCATACACGAACGGTCTCC-3'	not applicable

[T7]: T7 RNA Polymerase promoter sequence, 5'-TAATACGACTCACTATAGGG-3'
